# Obstetrics, and Diseases of Women and Children

**Published:** 1860-07

**Authors:** Wm. B. Atkinson

**Affiliations:** Lecturer on Obstetrics, and Diseases of Women and Children


### OBSTETRICS, AND DISEASES OF WOMEN AND CHILDREN.

BY WM. B. ATKINSON, M.D.,

LECTURER ON OBSTETRICS, AND DISEASES OF WOMEN AND CHILDREN.

### I.—*Alum in the Treatment of Uterine Hemorrhage.* By R. BEVERLEY COLE, M.D., San Francisco.

The double salt, the sulphate of alumina and potassa, has long been known as a powerful styptic. It has, in cases of hemorrhage, generally been employed in the form of powder taken by the stomach, or injection into the vagina. The writer asserts, in the most positive terms, that a crystal of alum of the size of a hen's egg, when introduced into the vagina, and carried to the os uteri, if retained, will *always* arrest the most obstinate hemorrhage. This assertion is made after six years experience in its use. Such is his confidence in this agent, that he never repairs to the bedside of a patient suffering from uterine hemorrhage, without first providing himself with a lump of alum, feeling every assurance that this, and no other remedy, will be required to arrest the flow. In employing this substance, it is important to select a firm crystal, which should be of such dimensions as, when introduced, to fill the vagina, and should be rendered smooth upon the surface by depriving it of its angles, and washing in water; in order to facilitate its removal, it may be perforated, and a string passed through the opening, by which it may be withdrawn. The length of time that it should be retained will depend upon circumstances, varying from thirty minutes to twenty-four hours.—(*San Francisco Med. Press*, January, 1860.)

II.—*Morning Sickness: its Significance as a Symptom.* By Thomas Inman, M.D., Liverpool.

Few, if any, understand the significance of this symptom, though all are familiar with its occurrence. A host of questions may be asked concerning it. Why are pregnant women sick? Why in the morning? Why in the early months more than the later? Why does not this phenomenon attend a distended bladder, or any other cause of enlargement of the abdomen, as frequently as it attends pregnancy? Does it occur in other complaints, and what have these in common with pregnancy? What is the proximate cause—is it to be sought in the stomach itself, the brain, the uterus, or all combined?

All pregnant women do not have it; some have it at one time, and not at another; it is rare among women in the country; and even among those who go from town, it is often found to cease, only to recur on returning to town. It is clear, then, that women are not sick because they are pregnant; something additional is required to produce the vomiting.

It is most common in the morning, yet it is not generally present so long as the recumbent position is maintained. As the brain is the organ most affected by change of posture, our attention is directed to that. Many cases are seen where vomiting occurs by assuming the erect position, and it is also a common sign of "water in the head." Few cases of this kind, however, have morning sickness; hence, a change in the cerebral circulation alone will not be sufficient to account for it.

We will next attempt to ascertain its causes by examining under what circumstances it occurs in males, children, and elderly people. In several cases of men suffering under this symptom, it was the result of dyspepsia. In children, it is found as a tolerably sure indication of deficiency of digestive power in the stomach and body generally. When we inquire how much the condition of the uterus influences the vomiting, we find that it is not produced solely by enlargement of that organ, for it is not common when the womb is distended by an accumulation in consequence of an unruptured hymen; nor is it from pressure in the pelvis, either direct or indirect, for it is generally absent in ovarian dropsy; nor is it a common sign in cases of uterine tumors, etc.

From all these considerations, we may infer that "uterine sympathy" does not hold so prominent a place as the formation of a new being. Nor can we assign to either the most important rank, as neither produces this phenomenon, unless other conditions are present.

As the symptom does not occur in perfectly healthy and strong women, we infer that its occurrence depends upon some deterioration of vital power, and as this involves more or less deterioration in all the organs, we infer that in such cases there is deficiency of vital power in the brain and stomach.

If this be true, the best remedies will be those which improve the general condition of the patient, which improve the steadiness of cerebral circulation, the tone of the stomach, and which deaden the sensibility of the organ which has been preternaturally increased by debility. If change of air cannot be adopted, then "diminish the daily work as far as possible, and increase the power to do it."

A case is related in illustration of this view, where a *man* had morning sickness for nine months every morning, about five minutes after rising. Regarding him as suffering from debility and overwork, he was ordered a glass of milk with some rum in it, to be taken before rising. Tincture of iron, and cod-liver oil were given through the day, with a nourishing diet and rest. The treatment was highly successful, and, after some four weeks, not having had any attacks of his old trouble, he one day worked too hard, and the next morning the sickness came on, and was again speedily relieved by the same treatment.

A similar plan must be adopted for pregnant women. They should take some food before rising, and allow sufficient time for this to have its influence. Perhaps milk combined with a stimulant is preferable; or hot coffee, cocoa, or tea. Where the sickness is very distressing, champagne or sparkling gooseberry wine answer well. Throughout the day, everything in the way of work must be carefully noted, in order that the patient may not overtask her powers. In bad cases, rest in bed may become necessary for a time.

Tonics, as steel, quinia, etc., are useful; opium, from its influence on the stomach and brain, is specially advantageous.

The preceding observations go far to explain those curious cases occasionally seen, where husbands suffer from this symptom as well as their wives; their sympathy being excited, soon produces faintness and sickness of the stomach. Such an explanation, however, will not apply to a case where a lady patient seriously asserted that in two out of four pregnancies, she was first made aware of her condition by the morning sickness of her husband. (*Brit. Med. Jour.*, March 24, 1860.)

III.—*Occlusion of the Os Uteri during Labor.* By J. C. METCALFE, M.D., Louisville, Ky.

The first case was aged thirty, well formed and healthy. She had complained for several days of irregular pains, and thought she had gone nearly three weeks over her time. An examination failed to detect any os, though it was evident an opening existed, as the liquor amnii was discharged. At a point corresponding to the left acetabulum there was a rugous feeling communicated to the touch, as if the uterus and vagina were irregularly attached; and the finger firmly pressed on this point caused considerable pain. The head of the fœtus could be felt through the walls of the uterus. About two years previous she was confined with her first child. The labor was tedious, a breech presentation, the head remaining in the pelvis from seven in the evening till five next morning, when it was extracted by a cotton hook inserted into the mouth, and traction by the feet. She was confined to bed about six weeks; very little pain, lochial discharge very offensive and profuse, and producing great prostration. The menses returned in about six months, and remained regular up to the time of conception.

As there was nothing in the case requiring immediate action, it was deemed best to wait. The next evening her condition was good; there was no change in the condition of the parts.

A speculum was now introduced, the parts were cleansed, and there was discovered, on close examination, among the rugæ, a small ulcer about the size of a three-cent piece, of an ashy color, which bled slightly at the touch. A probe passed readily through it into the cavity of the uterus, and was immediately followed by a discharge of matter resembling that from the small intestine, inodorous, mixed with what appeared to be healthy pus, with liquor amnii.

An artificial opening was made at the most dependent part, sufficient to admit two fingers. She was then replaced in bed, in the hope that active labor would come on. By next morning, being very weak, Dr. H. Miller was called in consultation. By his advice, the opening was much increased, and so profuse was the hemorrhage that the tampon became requisite. In spite, however, of this and the application of cold, etc., the discharge continued, and death ensued in about seven hours after the incisions were made. The autopsy disclosed the inferior half of the uterus filled with coagulæ surrounding the child. The pelvis was well formed, and of ordinary capacity. The incised parts were found to be nearly as thick as the body of the uterus. On the inner surface, corresponding to the rugæ on the vagina, was a firm ridge resembling a cicatrix.

The second case was aged forty-two, the mother of eight living children. Shortly after her eighth accouchment, she began to suffer from uterine disease, for which a caustic solution was applied. Under this treatment, she became much better. She was soon attacked with uterine hemorrhage, and passed a decomposed ovum. The discharge was very offensive, and was afterwards followed by a leucorrhœa, with much uterine suffering. About nine months after, Dr. Miller was summoned to attend her, for an attack of inflammation of the uterus, with fever of an intermittent type. She was now six months pregnant. Opium, quinia, and poultices were employed, but the pain continuing as if she were about to miscarry, Dr. M. was consulted. An examination was made, but no os could be found. About half an inch to the right of the axis of the pelvis, there was a transverse depression in the uterus about three-eighths of an inch long; below this was a slight oblong protuberance, resembling the inferior lip of the os uteri. No opening could be found. Two days after, as she was evidently sinking, in consultation with Dr. Miller, a transverse incision was made, from a very small opening at the depressed point. Through this enlargement, the fœtal head was felt. But little blood was lost. In the evening, she was restless, feverish, much prostrated. The pain less frequent, but of a more expulsive character; the head of the fœtus is firmly pressed against the opening and partially engages in it during the pain. In order to remove the fetid sanguineo-purulent discharge, the uterus was washed out with tepid water, and a stream of warm water played against the os, so as to relax it and increase the pain. This produced vertigo, and she sank into a state of stupor. Stimulants were given; and, lest death should ensue if she were not speedily delivered, a small pair of calculus forceps were introduced, and the delivery soon accomplished. Though exhaustion had come on, by the free exhibition of stimulants and heat, reaction was produced, and she made a good recovery.

Professor Henry Miller also related two cases, both of which were readily delivered after a similar operation to those above related, and soon recovered. Dr. Metcalfe thinks these cases were the result of some powerfully irritant action upon the parts, producing granulation, and subsequently a firm cicatrix. They should lead physicians to observe more care in the use of caustics, and other remedies which are calculated to produce occlusion of the os, and thus require the employment of the knife, perhaps to be followed by the death of the unfortunate patient.—(*Louisville Med. Jour.*, March, 1860.)

IV.—*Gangrene of the Uterus and Placenta, together with Intense Inflammation of the Peritoneum and half the Surface of the Child.* By G. W. PHILLIPS, M.D., St. Louis.

January 27, 1859, at 12 M., saw Mrs. H., who had had slight labor-pains for four hours previous to my seeing her. The membranes were protruding through the os, and very intense. The patient had a constant retching, and complained of great soreness over the whole abdomen. After three hours had passed, without any change in the symptoms, the membranes were ruptured, and a large quantity of water discharged. On introducing the whole hand, the ear of the child was detected just above the pelvic brim, the head lying crossways, the occiput to the right ilium, the head and neck doubled up on the shoulder. After vainly endeavoring to rectify the position, a small quantity of ergot was given without effect. The soreness of the abdomen rapidly increased, and, to check the inflammatory symptoms, she was bled sixteen ounces, and had one-third of a grain of morphia. This eased her, but the vomiting continued. No progress was made in the delivery, the pain was renewed, and death took place at 4 P.M. On making a post-mortem, the child was found in the position above mentioned, the whole of the placenta, and a great part of the uterus being in a state of mortification. The peritoneum was intensely inflamed, as also one-half of the child's body.—(*St. Louis Med. and Surg. Jour.*, May, 1860.)

V.—*Rupture of the Uterus, and Ventral Hernia during Pregnancy.* By M. BOURGEOIS, Paris.

A woman whose inlet of the pelvis measured only two inches and a half, and who had the Cæsarean operation performed, became pregnant one year afterwards. At the fourth month she had severe abdominal pain and vomiting. On the next day she noticed a peculiar tumor at the lower portion of the abdomen, and in the median line. The pain and vomiting continued for a week, with occasional intermissions. On examination, M. Bourgeois found a globular tumor projecting immediately under the skin of the abdomen, of the size of the head of a full-grown fœtus. When the patient stood upright, the tumor fell forward over the pubis like a sac, and was herniated between the muscles of the abdomen. When she lay down, the mass could be returned under the muscles. By the hand being placed on the attenuated skin, the prominent parts of a fœtus could be made out; the feet could be seized one after the other, and followed up to the knees, and it was easily felt that the fœtus was separated from the skin only by the resisting and globular uterus. The child did not evince any life when tried by auscultation. M. Bourgeois considered that the fœtus had left the uterus, and that hernia of the linea alba existed. A belt was ordered, to keep up the mass; and the patient soon improved by keeping her bed, and using a very simple line of treatment. Six weeks afterwards labor-pains came on, and uterine hemorrhage took place, fetid clots being expelled; severe peritonitis set in, which was allayed by belladonna and calomel. The hemorrhage continued, and the discharge from the vulva was observed up to the seventh month of gestation, when it was very abundant, but the patient free from pain. The abdomen became smaller, and the ventral hernia less marked. At the eighth month the fetid discharge was very slight.

At this period a committee was appointed by the Surgical Society of Paris to examine the patient, and decide as to the treatment to be adopted. They considered that no active intervention should be resorted to, and that the fœtus probably retained a certain amount of connection with the uterus. The termination has not yet been published.—(*Lancet*, March 31, 1860.)

VI.—*Displacements of the Uterus.* By E. R. Peaslee, M.D., New York.

The general indications are to replace the uterus, and to maintain it in its normal position by such aids as may be either temporarily or permanently required.

The first is accomplished by manipulations alone, or with instruments; the second by various means, the most prominent of which are astringents and mechanical support. Of astringents, the tanic acid, alum, sulphate of zinc, etc. are used. Some writers regard these as injurious, and they will do harm if injudiciously employed. Dr. Hamilton thinks that most of the cases of chronic enlargement of the uterus seen by him were produced by the employment of injections.

They will often be found of advantage, if used in accordance with the following rules:—

Withhold them entirely if there is any inflammation, or even much congestion of the vagina or uterus.

Use them of a very weak strength at first.

In case of a profuse discharge, never check it suddenly by their use.

Discontinue the application if any bad effects ensue from it.

Used with these precautions, astringent vaginal applications are valuable aids in the treatment of displacements, as well as other diseases of the uterus.

Mechanical supports are either internal or external. The latter only can be useful to support the walls of the abdomen in cases of relaxation, and may even be an exciting cause of displacement; hence they should be employed with extreme caution.

The internal supports are generally known as "pessaries." Thus, we have a ball of lint soaked in an astringent solution, or a muslin bag filled with an astringent powder, or instruments of a variety of shapes, made of wood, glass, ivory, etc.

By some these are objected to as being indelicate, causing a vaginal discharge; if too small, being of no use; if too large, producing dilatation of the vagina, causing ulceration, and contraction of the vagina; becoming incrusted with calcareous matter, etc.

If but a small part of these effects of the use of pessaries be unavoidable, very few practitioners would use them. But this is by no means the fact, and to denounce an instrument because it may be made to do harm is unphilosophical.

They are of service if used in accordance with the following rules:—

Never employ a pessary if there be irritation or inflammation of the vagina or cervix uteri. Adapt the size precisely to the case. Watch the patient, and remove the instrument if occasion arise for so doing. Examine at proper intervals to see that no mischievous effects are produced. Teach the woman to keep the instrument clean by daily vaginal injections of water or weak soapsuds. Remove the pessary occasionally to see that it remains in good condition.

Again, a pessary should be as light as possible; it should be made of a material which will retain a smooth surface, and therefore not be acted on by the fluids, and of a form to cause the least possible pressure consistent with securing the object for which it is used. The lightest form is the ring, and hence is preferable. This should be made of silver or gold, as glass or ivory are too heavy. Gutta-percha, if used in its purity, is excellent. This form may be made of a piece of watch-spring, fastened in the shape of a ring, and then covered with the gum.

The *globe* pessary is sometimes of importance to aid in replacing and then retaining the uterus.

Stem pessaries, and those made of cork, sponge, etc. are highly objectionable for the reasons above named.

"Of late, therefore, I have confined myself, with very rare exceptions, to the ring pessary made of gutta-percha or metal, and the globe pessary, made in the form of a hollow sphere, of silver or gold."—(*Am. Med. Monthly*, April, 1860.)

VII.—*Puerperal Convulsions successfully treated by Subcutaneous Injections of Morphia.* By PROF. SCANZONI, of Wurtzburg.

Prof. Scanzoni has long employed this method of treating disease with success, but attaches especial importance to the following case, as it seems to prove that the subcutaneous application of narcotics furnishes a means of acting on abnormal irritations of the brain with greater rapidity and certainty than by the mouth. Prof. S. is convinced that a kind of intoxication produced by opium leads with more certainty to a favorable termination than any other remedies for this terrible disease. But, as from a variety of causes, it is not always possible to employ this agent either by the mouth or per rectum, the subcutaneous injection of these remedies supplies a means by which to overcome these difficulties.

He is also convinced by numerous experiments that, although the effect of this method is not always persistent, yet it is constantly produced, and that within a very short time after the injection. The symptoms of the action of the remedy upon the brain are drowsiness, giddiness, headache, sickness, feeling of constriction in the throat, even vomiting and depression. These facts, taken along with the known effects of the subcutaneous application in delirium tremens, mania, chorea, tetanus, etc., induced him to try the same treatment in puerperal convulsions, and with the most satisfactory results. After three injections of meconate of morphia, there occurred only two attacks in nine hours, while previously there had been three attacks in an hour and three-quarters. This is the more remarkable, as experience has shown that, as a general rule, the paroxysms became not only more violent, but follow at shorter intervals as the labor advances.

*Case D*, aged twenty-one, primipara, strong and robust, seized with nervous paroxysms and loss of consciousness in the night of June seventh. The whole body and extremities œdematous; sounds of the fœtal heart distinct; os uteri dilated to the size of a sixpence; head presenting. Urine very albuminous. At eight o'clock next day she was seized with a second convulsive attack of a marked character, which lasted for some minutes. On recovering consciousness, she could answer questions. A third convulsion succeeded at a quarter to nine, a fourth in an hour; again in two hours, and again at six. After the fourth, consciousness did not return, and the breathing become stertorous. At ten o'clock, she was bled about eight ounces; an enema, with twenty-five drops of laudanum, was given; the body was put into a warm bath, while cold was applied to the head. The meconate of morphia was now, at three different times, injected under the skin, the quantity amounting in all to about ten grains of opium. Labor advances slowly. At three o'clock next morning the membranes burst; the os dilated to the size of a half-crown; the head still high up above the brim; sounds of the heart very distinct. At seven, the os was larger than a crown-piece, very dilatable, the head high up and immovable; complete loss of consciousness, profound coma. The forceps were now employed, and a live child delivered without much difficulty; the placenta followed. Some wine, and ten drops of tincture of amber and musk were now given. At eleven, a seventh attack came on, but was slight and short; after which she became excited, but toward morning grew more calm. At nine in the morning, she could answer questions, and remained like a drunken person; pulse 128. Nothing but lemonade now given. During the night there were several slight attacks of mania. In the morning she answered rationally; pulse 108; œdema diminished, abdomen tender, and numerous râles in the lungs. Warm bath, lemonade, and expectorants were prescribed. Evening, quite herself; pulse 132. She went on well, and by June seventeenth all albumen had disappeared from the urine, and by the twenty-first she left the hospital with her child, being advised to continue the use of steel for a considerable time.—(*Bull. Gen. de Thérap.*, March, 1860; *Ed. Med. Jour.*, May, 1860.)

VIII.—*Collyrium in the Ophthalmia of New-born Infants.* By M. FOUCHER.

Glycerin, fʒ ix; Argenti Nitrat. gr. jss ad iij. The eye is first washed by injecting a very weak solution of chloride of sodium, and then a drop of the above is deposited on the internal surface of the eyelids by means of a small camel's-hair pencil.—(*Louisville Med. Jour.*, March, 1860.)

